# Effectiveness and Safety of Non‐Surgical Otoplasty Using APTOS Threads: A Retrospective Study

**DOI:** 10.1111/jocd.70581

**Published:** 2025-12-10

**Authors:** Haidar Nabeeh Algousous, Mariam Nikolaishvili, Marlen Sulamanidze

**Affiliations:** ^1^ AL‐GOUSOUS Medical Center Amman Jordan; ^2^ School of Natural Sciences and Medicine Ilia State University Tbilisi Georgia; ^3^ Clinic of Plastic and Aesthetic Surgery and Cosmetology “Total Charm Orbeliani” Tbilisi Georgia

**Keywords:** barbed threads, ear correction, minimally invasive otoplasty, non‐surgical otoplasty, prominent ears correction

## Abstract

**Importance:**

Prominent ear deformities can negatively impact self‐esteem and social well‐being. Surgical otoplasty is effective but is associated with complications, longer recovery, and altered ear contours.

**Objective:**

To evaluate the effectiveness and safety of non‐surgical otoplasty using Aptos threads for the correction of prominent ear deformities and other auricular deformities.

**Design:**

Retrospective observational study.

**Setting:**

Single‐center study conducted at AL‐GOUSOUS Medical Center, Amman, Jordan.

**Participants:**

A total of 250 patients (500 ears) aged 7–67 years (mean age: 30.6 ± 12.9 years), including 41 participants under 18. Inclusion criteria were prominent ears, asymmetry, congenital deformities, or complications from previous surgery. Exclusion criteria included pregnancy, keloid history, skin disorders, infections, or allergy to thread materials.

**Intervention or Exposures:**

Thread‐based otoplasty using the Aptos Sole Otoplasty Kit under local anesthesia, guided by pre‐marked Π‐shaped and U‐shaped paths along the auricular cartilage.

**Main Outcome Measures:**

Patient satisfaction was assessed via EAR‐Q modules (Appearance, Appearance Distress, Psychological and Social Function), and safety was evaluated using the EAR‐Q Adverse Effects module. Aesthetic outcomes were evaluated with the Global Aesthetic Improvement Scale (GAIS). Follow‐ups were conducted at 1 month and 1 year.

**Results:**

Significant improvements were observed in all EAR‐Q domains from baseline to 1 month and 1 year (*p* < 0.001), with the Appearance Distress score continuing to improve between the 1‐month and 1‐year follow‐up. GAIS scores indicated exceptional or very good aesthetic results in 90% of patients at 1 month and 87% at 1 year. Adverse effects were minimal, with no reports of hematoma, infection, or scarring. Pain and numbness resolved by 1 year post‐procedure.

**Conclusions:**

Aptos thread‐based otoplasty is a safe and effective alternative to surgery for correcting prominent ears and auricular asymmetry, offering high patient satisfaction, minimal complications, and natural aesthetic results.

**Relevance:**

This non‐surgical approach offers a reliable, minimally invasive treatment option for patients seeking ear correction without the risks and downtime of traditional otoplasty.

## 1 Introduction

Prominent ear, one of the most common ear deformities, affects approximately 5% of the population, typically occurring bilaterally [1]. It is an autosomal dominant condition seen more frequently in Caucasians [2]. Both genetic and environmental factors, such as postpartum forces, may contribute to its development. The deformity results from various anatomical abnormalities, including underdevelopment of the antihelix, hypertrophy or anterior rotation of the conchal cartilage, and a poorly defined helical rim [3]. Although this condition has no functional implications, children with protruding ears may face social challenges, including teasing or bullying, which can lead to psychological distress. In cases where the condition significantly impacts a child's well‐being, otoplasty may be considered as a treatment option, typically performed between the ages of 4 and 6 [4]. The diagnosis of prominent ears is often subjective. While there are many methods to measure the size and angles of the ear, there is no clear standard that defines what is considered “normal” or “prominent,” making it difficult to have a precise definition [5]. The negative social and emotional effects of prominent ears are often the main reason patients seek surgical correction [6]. Otoplasty for correcting prominent ears is a widely accepted cosmetic procedure in the population. It is performed primarily for aesthetic reasons rather than to address any functional or occupational issues. Various methods for correcting prominent ears exist, but most of them involve surgery, which often leads to pain and a lengthy recovery period. While the outcomes are generally satisfactory for both patients and surgeons, many techniques can compromise the natural contours of the ear. Procedures involving cartilage scoring or excision frequently cause sharp or jagged edges on the antihelix and may destabilize the cartilage. During healing, this can lead to unnatural step‐offs due to the altered tension in the ear's structure [7].

Considering the challenges associated with traditional surgical methods for otoplasty, we conducted a study to evaluate the effectiveness of a non‐surgical approach using Aptos threads. This study aimed to assess the aesthetic outcomes, patient satisfaction, and complication rates of thread‐based otoplasty. A total of 250 patients (500 ears) were treated using Aptos non‐resorbable threads, addressing conditions such as prominent ears, asymmetry, and congenital deformities. Our study offers valuable insights into the potential benefits of this minimally invasive technique.

## 2 Materials and Methods

### 2.1 Patient Selection

The study included 250 patients, 500 ears were treated. Eligibility criteria required patients to have at least one of the following: prominent ears, ear asymmetry, congenital deformities, overcorrection from prior surgery, protruding ears resulting from trauma, or a desire for earlobe reduction, reshaping, or aesthetic improvement. Patients were not included if they met any of the following criteria: pregnancy or breastfeeding, a history of keloid or hypertrophic scar formation, prior ear surgeries unrelated to otoplasty, concurrent dermatological or autoimmune disorders, infection in the treatment area, or a known allergy to the thread material.

### 2.2 Procedure Details

#### 2.2.1 Thread Implantation

The Sole Otoplasty kit by Aptos was used. It included one polypropylene thread (USP 4/0, EP 1.5) with a length of 800 mm. The kit also comprised a trocar point needle (DRT 1.2 × 50 mm, 3/8 circle) and a standard needle attachment.

### 2.3 Procedure Description

#### 2.3.1 Marking

**FIGURE 1:**
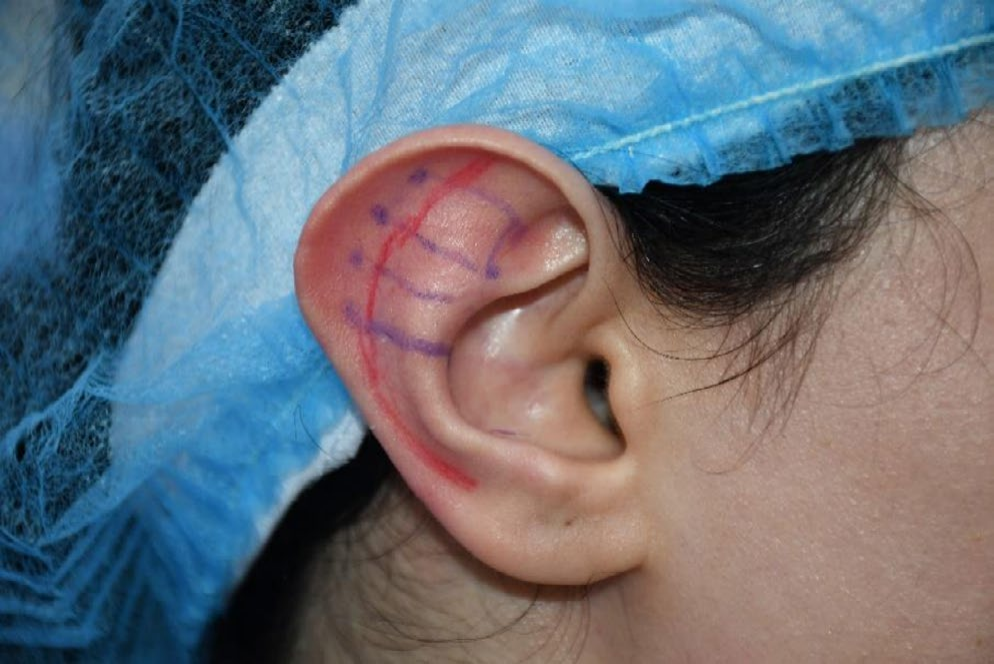
Marking for thread insertion.

The marking technique for this procedure resembled the Mustarde otoplasty technique for antihelix correction [8]. Two or three П‐shaped paths were marked transversely on the anterior surface of the antihelix. The number and placement of these marking lines were determined individually for each case (Figure 1).

#### 2.3.2 Anesthesia

Infiltration anesthesia was administered in the incision area using a 30G needle. Further anesthesia was administered along the previously drawn marking lines. Minor tissue hydro‐preparation of both the anterior and posterior surfaces of the auricle was performed to facilitate the procedure. The optimal anesthetic volume was approximately 5 mL per side.

#### 2.3.3 Procedure

A small skin incision (~0.3 cm) was made on the posterior auricular surface at the antihelix projection. A polypropylene thread (Aptos Needle 4/0) was inserted along the pre‐marked U‐shaped path toward the anterior surface. The needle exited partially, then advanced subdermally along the antihelix and redirected through the cartilage to the posterior surface. The process was repeated through successive П‐shaped paths. The thread was tightened to form the desired antihelix contour, tied with a surgical knot, buried under the skin, and the incision was closed with 5/0 or 6/0 sutures. Additional paths were treated similarly if needed.

#### 2.3.4 Post‐Procedure Care

Due to minimal subcutaneous tissue, the thread was guided entirely subdermally. After disinfection and upright evaluation, mild asymmetry, swelling, or redness was expected to resolve within 2 weeks. A compression bandage was applied and recommended for 7–10 days to support the results.

### 2.4 Evaluation and Statistical Analysis

Patient's satisfaction was evaluated using four scales of the validated EAR‐Q questionnaire (Copyright 2020, McMaster University and The Hospital for Sick Children)—Appearance of the Ears, Appearance Distress, Psychological Function, and Social Function—along with safety assessment with the EAR‐Q module for adverse effects. These EAR‐Q questionnaires measure specific aspects of patient‐reported outcomes following ear‐related interventions:

- The Appearance of the Ears assesses patient satisfaction with the appearance of their ears through 10 questions, each scored on a 4‐point scale ranging from 1 (not at all) to 4 (very much).
- The Appearance Distress evaluates the emotional impact of ear appearance using 8 questions. Responses are recorded on a 4‐point scale, from 1 (always) to 4 (never).
- The Psychological Function measures general psychological well‐being with 10 questions. Each response is rated on a 4‐point scale, from 1 (never) to 4 (always).
- The Social Function assesses social interactions and acceptance through 10 questions. Responses are recorded on a 4‐point scale, from 1 (never) to 4 (always).
- The Adverse Effects examines the occurrence of common adverse events associated with otoplasty through 10 questions, with responses scored on a 3‐point scale from 1 (a lot) to 3 (not at all).

The EAR‐Q questionnaires were administered at three time points: pre‐procedure, 1 month (short‐term assessment) and 1 year post‐procedure (long‐term assessment) with the exception of the EAR‐Q Adverse Effects module which was used only post‐procedure. Within each module responses to each individual question were analyzed as well as total scores. A repeated measures ANOVA was used to evaluate the overall statistical significance of changes in EAR‐Q responses over time. Paired t‐tests were used for pairwise comparisons across three time points: baseline, 1 month, and 1 year. Bonferroni adjustment was applied to control for Type I error due to multiple comparisons. Statistical analysis was performed using Microsoft Excel and R Statistical Software [9].

The Global Aesthetic Improvement Scale (GAIS) for Investigator was employed for assessment of overall aesthetic outcomes by the doctor 1 month and 1 year after thread implantation. GAIS is a validated subjective assessment method that incorporates five response options, reflecting the degree of perceived aesthetic improvement assessed separately by the subject and the doctor: (1) exceptional improvement, (2) very improved patient, (3) improved, (4) unaltered patient, (5) worsened patient.

## 3 Results

### 3.1 Study Subjects

A total of 250 patients were enrolled in a study: 109 males (43.6%) and 141 females (56.4%), aged 7–67 years. Mean (±SD) age was 30.6 ± 12.9 years; 41 subjects (16.4%) were below 18 years. The primary reasons for seeking ear correction were prominent ears (23.6%), aesthetic enhancement (17.6%), congenital deformities (16.4%), asymmetrical ears (13.2%), overcorrected previous surgery (10.8%), earlobe reduction or reshaping (9.2%), and protruding ears following trauma (8.4%).

### 3.2 Effectiveness of Treatment

#### 3.2.1 EAR‐Q: Appearance of the Ears

**FIGURE 2:**
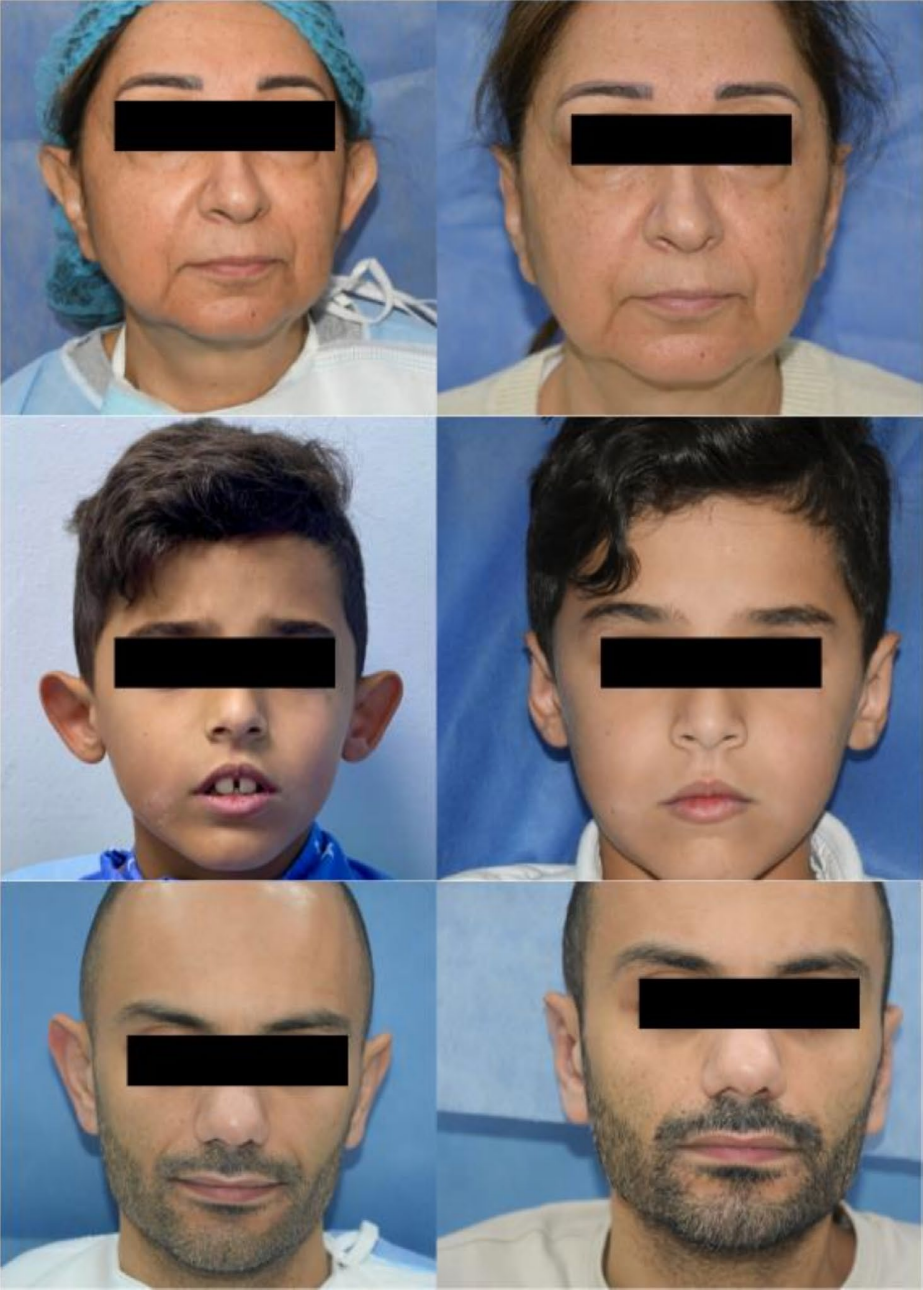
Before the procedure (left) and 1 year after the procedure (right).

The mean values (±SD) for Appearance of the Ears score were 18.7 ± 6.1, 33.4 ± 5.3, and 36.2 ± 3.3 at baseline, 1 month and 1 year after thread implantation respectively (Table 1). At baseline, responses varied across the questions. However, a dramatic shift in satisfaction was observed at the one‐year mark, with nearly all participants selecting one of the high‐scoring options (Table 2). Mean scores for answers to each question of the module are presented in Figure 2.

**TABLE 1:** Mean total scores for responses to EAR‐Q modules at baseline, 1 month and 1 year after treatment.

Questionnaire module	Baseline	1 month	1 year
	Total score, mean ± SD		
Appearance of the ears^a^	18.7 ± 6.1	33.4 ± 5.3*	36.2 ± 3.3*
Appearance distress^a^	15.5 ± 5.1	27.2 ± 4.0*	29.7 ± 3.6*
Psychological function	23.9 ± 8.1	32.7 ± 5.6*	35.5 ± 3.9*
Social function^a^	23.8 ± 6.9	31.6 ± 4.7*	33.9 ± 4.1*
Ear adverse effects	—	29.1 ± 0.9	29.6 ± 0.5

Abbreviation: SD, standard deviation.

^a^ Statistically significant main effect of time, repeated measures ANOVA, *p* < 0.001.

* *p* < 0.01 compared to baseline, paired *t*‐test with Bonferroni correction.

**TABLE 2:** Ear‐Q appearance of the ears, proportion of subjects with scores 3 (“quite a bit”) and 4 (“very much”) in response to the question “how much do you like…?”.

	Question	Score	% of answers, baseline	% of answers, 1 month	% of answers, 1 year
How much do you like…	How your ears look from far away?	Quite a bit	18	28	10
		Very much	0	67	90
	The overall shape of your ears?	Quite a bit	18	42	42
		Very much	0	48	58
	The size of your ears?	Quite a bit	32	42	42
		Very much	4	53	58
	How your ears look when your hair is wet?	Quite a bit	28	44	38
		Very much	0	47	62
	How the top part of your ears look?	Quite a bit	27	34	38
		Very much	0	47	58
	How your ears look in photos?	Quite a bit	46	32	14
		Very much	4	68	86
	How your ears look from the side (your profile)?	Quite a bit	32	62	43
		Very much	0	24	57
	How your ears look if you put on a hat that shows your ears (e.g., a baseball cap)?	Quite a bit	19	52	62
		Very much	0	29	38
	How your ears look up close?	Quite a bit	10	43	43
		Very much	5	33	57
	How your ears look compared with other people's ears?	Quite a bit	0	48	38
		Very much	0	43	62

The ANOVA analysis revealed a statistically significant main effect of time on the total Appearance of the Ears score (*F*(2, 744) = 29.75, *p* < 0.001, repeated measures ANOVA). The within‐participant residual variance (Mean Squared Error [MSE] = 642) was relatively small compared to the variance explained by the time factor (Mean Sum of Squares [MSS] = 19 116), indicating that a significant proportion of variability is attributable to changes over time rather than random variation. Pairwise timepoint comparison showed that there was no significant difference between Appearance of the Ears scores 1 month and 1 year after implantation (*p* = 0.72). However, the scores 1 month and 1 year after implantation were statistically significantly higher than baseline (*p* < 0.001 for both).

#### 3.2.2 EAR‐Q: Appearance Distress

**FIGURE 3:**
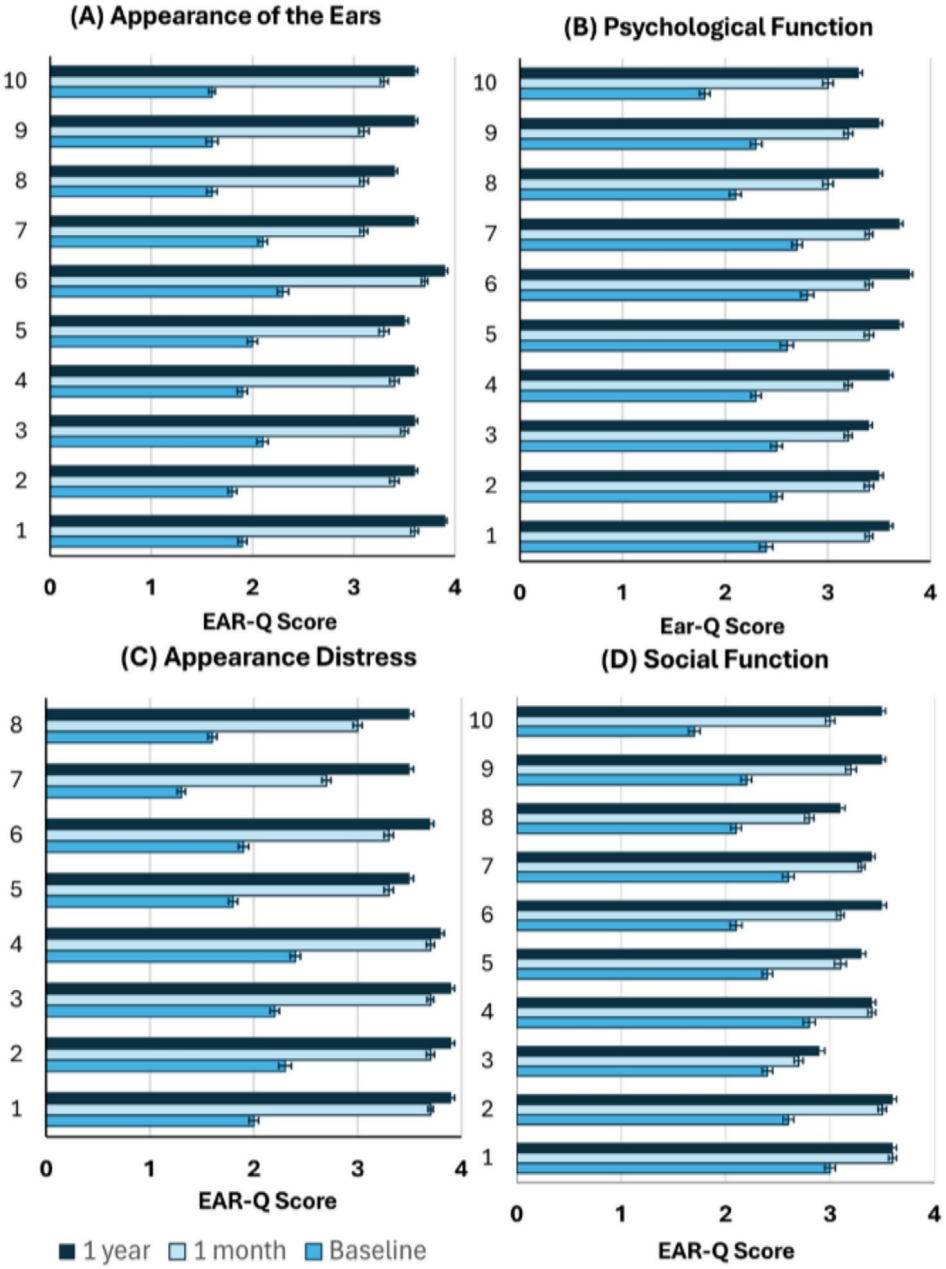
Mean scores (±SEM) for responses to each question at baseline, 1 month and 1 year after treatment. (A) Appearance of the ears module of EAR‐Q‐ Questions: 1. How your ears look compared with other people's ears?; 2. How your ears look up close?; 3. How your ears look if you put on a hat that shows your ears (e.g., a baseball cap?); 4. How your ears look from the side(your profile)?; 5. How your ears look in photos?; 6. How the top part of your ears look?; 7. How your ears look when your hair is wet?; 8. The size of your ears?; 9. The overall shape of your ears?; 10. How your ears look from far away? Scoring for responses: 1—Not at all, 2—A little bit, 3—Quite a bit, 4—Very much. (B) Psychological Function module of EAR‐Q‐Questions: 1. I feel good about how I look; 2. I feel great about myself; 3. I feel confident; 4. I like myself; 5. I am proud of myself; 6. I believe in myself; 7. I feel okay about myself; 8. I feel happy; 9. I enjoy life; 10. I am happy with my life. Scoring for responses: 1—Never, 2—Sometimes, 3—Often, 4—Always. (C) Appearance Distress module of EAR‐Q‐ Questions: 1. I feel Self‐conscious about how I look; 2. I get upset when people stare at me; 3. I dislike how I look; 4. I feel unhappy about how I look; 5. It's hard to meet new people because of how I look; 6. I cover up or hide how I look when I go out; 7. I avoid looking at myself in a mirror; 8. I avoid going out because of how I look (like to a party). Scoring for responses: 1—Always, 2—Often, 3—Sometimes, 4—Never. (D) Social Function module of EAR‐Q‐Questions: 1. it's okay when people look at my face; 2. I feel the same as other people my age; 3. It's easy for me to make friends; 4. I feel like I fit in; 5. I feel confident when I go out (like to a party); 6. I like being with other people; 7. People treat me the same as everyone else; 8. People listen to what I have to say; 9. I have fun with friends; 10. My friends accept me Scoring for responses: 1—Never, 2—Sometimes, 3—Often, 4—Always.

The mean values for Appearance Distress score were 15.5 ± 5.1, 27.2 ± 4.0, and 29.7 ± 3.6 at baseline, 1 month and 1 year after thread implantation respectively (Table 1). At baseline, for 6 of 8 questions of the module, none of the participants selected the answer “Never,” with the majority choosing “Always” or “Often”. By 1 year, this pattern had reversed, with most responses being “Never” and none selecting “Always” or “Often”. Mean scores for answers to each question of the module are presented in Figure 3.

The repeated measures ANOVA analysis revealed a statistically significant main effect of time on the total Appearance Distress score (*F*(2, 744) = 179.6, *p* < 0.001). The MSS = 26 430 was substantially larger than the MSE = 147, supporting the effect of time on the measured outcome.

Pairwise timepoint comparison with paired t‐test revealed that scores 1 month and 1 year after implantation were statistically significantly higher than at baseline (*p* < 0.001 for both). Scores at 1 year were also statistically significantly higher than 1 month after implantation (*p* < 0.001).

#### 3.2.3 EAR‐Q: Psychological Function

The mean values for Psychological Function score were 23.9 ± 8.1, 32.7 ± 5.6, and 35.5 ± 3.9 at baseline, 1 month and 1 year after thread implantation respectively (Table 1). At baseline, subjects' responses varied across the questions. However, by one month post‐treatment, none of the participants selected “Never.” By 1 year, 8 out of 10 questions were answered exclusively with “Always” or “Often.” Mean scores for answers to each question are given in Figure 3.

The repeated measures ANOVA analysis did not reveal a statistically significant main effect of time on the psychological function (*F*(2, 744) = 2.6, *p* = 0.075); however, the pairwise *t*‐test showed that scores at baseline were significantly lower than at 1 month (*p* = 5.5 × 10^−6^) and 1 year (*p* = 0.0018) after implantation. Scores at 1 month and 1 year were not statistically significantly different.

#### 3.2.4 EAR‐Q: Social Function

The mean values for Social Function score were 23.8 ± 6.9, 31.6 ± 4.7, and 33.9 ± 4.1 at baseline, 1 month and 1 year after thread implantation respectively. At baseline, subjects' responses to questions of this module were various. By 1 year, none of the participants selected “Never” and most answers were “Often” and “Always”. Mean scores for answers to each question are given in Figure 3.

The repeated measures ANOVA analysis revealed a statistically significant main effect of time on the total Social Function score (*F*(2, 744) = 37.23, *p* < 0.001). MSS = 9317 was substantially larger than the MSE = 250, suggesting that the observed variation over time was not due to random fluctuations.

Paired *t*‐test showed no significant difference between Social Function scores at 1 month and 1 year. The scores 1 month and 1 year after implantation were statistically significantly higher compared to baseline (*p* < 0.001 for both).

#### 3.2.5 EAR‐Q: EAR Adverse Effects

The mean Ear Adverse Effects scores were similar at 1 month and 1 year after thread implantation: 29.1 ± 0.9 and 29.6 ± 0.5 respectively with no statistically significant difference between them. None of the subjects answered that any of the adverse effects occurred “A lot”, percentages of answers “A little bit” and “Not at all” are presented in Table 3. None of the participants experienced bruising, discoloration, or swelling at either time point. Adverse effects such as pain while sleeping on the side and ear itching, which were more commonly reported occurring one month post‐treatment, significantly decreased by the one‐year mark.

**TABLE 3:** Percentage of subjects who reported adverse effects by giving the answer “A little”, or “Not at all” to questions of the EAR‐Q Ear Adverse Effects questionnaire one month and one year after otoplasty.

Question	Answer	% of responses, 1 month	% of responses, 1 year
There is blood or other fluid coming from my ear scars	A little	0	0
	Not at all	100	100
My ears feel tingly (pins and needles feeling)	A little	18.7	18.7
	Not at all	81.3	81.3
My ears hurt when I am active (e.g., run, jump)	A little	9.2	0
	Not at all	90.8	100
My ears are bruised	A little	0	0
	Not at all	100	100
My ears look discolored (a different color from usual)	A little	0	0
	Not at all	100	100
My ears are puffy or swollen (bigger than normal)	A little	0	0
	Not at all	100	100
My ears feel numb (cannot feel them)	A little	14.3	4.8
	Not at all	85.7	95.2
My ears hurt when I sleep on my side	A little	23.9	4.8
	Not at all	76.1	95.2
My ears feel itchy	A little	19.1	9.6
	Not at all	80.9	90.4
My ears are sensitive when I touch them	A little	4.8	4.8
	Not at all	95.2	95.2

Overall, no complications were reported up to 1 month after the procedure.

Thus, the effectiveness of Aptos threads was supported by a statistically significant improvement in subjects' satisfaction with their ears after thread implantation. This is confirmed by a statistically significant increase in total scores for Appearance of the Ears, Appearance Distress, Psychological Function and Social Function after thread implantation. The safety of the intervention was supported by the results from the Ear Adverse Effects questionnaire. The effect was maintained and even improved 1 year after implantation.

#### 3.2.6 Global Aesthetic Improvement Score

GAIS assessment was performed by the doctor at follow‐up visits, for both ears. The mean (±SD) GAIS scores were 1.4 ± 0.7 and 1.5 ± 0.7 1 month and 1 year after thread implantation respectively. The subject distribution over GAIS scores assigned by the doctor, is shown in Table 4. None of the study participants had unaltered or worsened ears in the doctor's opinion, and most subjects received the “exceptional improvement” assessment both 1 month and 1 year after implantation.

**TABLE 4:** GAIS assessment by the doctor 1 month and 1 year after implantation.

GAIS assessment	1 Month (%)	1 Year (%)
Exceptional improvement	70	64
Very improved	20	23
Improved	10	13
Unaltered	0	0
Worsened	0	0

Examples of ear improvement are given in Figure 2.

## 4 Discussion

Thread lifting has emerged as a widely favored aesthetic procedure due to its effective lifting capabilities and significantly reduced downtime compared to traditional surgical otoplasty. Non‐surgical cosmetic interventions are increasingly in demand, particularly among patients with busy lifestyles who prefer treatments that offer immediate results with minimal complications and negligible social downtime.

This study is one of the first to provide clinical evidence of ear improvement using lifting threads. It demonstrated statistically significant improvement in subjects' satisfaction with the aesthetic appearance of their ears and their psychological well‐being as well as good safety outcomes. According to the systematic review by Sadhra et al., complications such as pain (13%), hematoma (2.5%), infection (0.8%), scarring (1.6%), revision surgeries (5%) and wound healing problems (3%) are commonly associated with surgical otoplasty [10]. These complications, while generally infrequent, can significantly impact patient satisfaction and outcomes. In this study, the thread‐based otoplasty demonstrated minimal adverse effects with low occurrence of pain during activities at 1 month (9.2%) which resolved entirely by 1 year. Numbness, reported in 14.3% of patients at 1 month in our study, decreased to 4.8% at 1 year, suggesting progressive sensory recovery. Notably, complications such as infections or the need for revision surgeries, assessed at the level of 5% in surgical otoplasty by Sadhra et al., were entirely absent with the thread‐based approach. Thus, the study supports the reduced risk profile of thread otoplasty while maintaining favorable aesthetic and functional outcomes.

This retrospective study has several limitations inherent to its design, including the absence of a comparator group, its single‐center nature, and the potential impact of uncontrolled confounding factors. These limitations underscore the need for a future prospective trial to validate these findings and provide a more thorough understanding of the long‐term safety and efficacy of lifting threads for otoplasty. Furthermore, incorporating objective measurements alongside subjective outcome assessments in future studies will enhance the reliability and robustness of the results.

The overwhelmingly positive feedback from patients further supports the adoption of thread lifting as an effective, minimally invasive approach to otoplasty. This technique offers similar aesthetic and psychological benefits as surgical otoplasty, without the associated drawbacks, making it an attractive option for patients seeking reliable, safe, and convenient solutions to improve the appearance of their ears.

## 5 Conclusion

This study demonstrates that thread‐based otoplasty using the Aptos Sole Otoplasty Kit is a safe and effective method for addressing prominent ear deformities and other auricular conditions. Significant improvements were observed in ear appearance, psychological well‐being, and social function, as measured by the EAR‐Q questionnaire, with high levels of aesthetic satisfaction confirmed by GAIS scores. Adverse events were minimal, self‐limited, and resolved without intervention. Despite the study's limitations, the findings support the safety and efficacy of this.

## Funding

The authors have nothing to report.

## Ethics Statement

This study was conducted at Algousous Medical Center. Informed consent was obtained from all participants prior to inclusion in the study. For participants under the age of 18, consent was provided by their parents or legal representatives.

## Conflicts of Interest

The authors declare no conflicts of interest.

## Data Availability

The data that supports the findings of this study are available from the corresponding author upon reasonable request.
